# The elephant in the room: Intimate partner violence, women, and traumatic brain injury in sub-Saharan Africa

**DOI:** 10.3389/fneur.2022.917967

**Published:** 2022-09-06

**Authors:** Martina Anto-Ocrah, Richard Gyan Aboagye, Linda Hasman, Ali Ghanem, Seth Owusu-Agyei, Raquel Buranosky

**Affiliations:** ^1^Division of General Internal Medicine, Department of Medicine, University of Pittsburgh, Pittsburgh, PA, United States; ^2^Department of Family and Community Health, Fred N. Binka School of Public Health, University of Health and Allied Sciences, Hohoe, Ghana; ^3^Institute for Innovative Education: Miner Libraries, University of Rochester Medical Center, Rochester, NY, United States; ^4^Institute for Global Health, University College London, London, United Kingdom; ^5^Institute of Health Research, University of Health and Allied Sciences, Hohoe, Ghana

**Keywords:** traumatic brain injury, Africa, intimate partner violence (IPV), biomarkers, HPA, neuroimaging, gender, social determinants of health

## Abstract

**Background:**

Intimate partner violence (IPV) is a gendered form of violence that has been linked with traumatic brain injury (TBI). The prevalence of IPV in sub-Saharan Africa (SSA) is estimated to be one of the highest globally. Yet, little is known about the association between IPV and TBI in the SSA context. In this scoping review, we examine the intersection between IPV and TBI in SSA to identify gaps, as well as intervention opportunities.

**Methods:**

Using the Preferred Reporting Items for Systematic Reviews and Meta-Analyses—Extension for Scoping Review (PRISMA-ScR) guidelines to guide our analyses and reporting, we searched for published articles indexed in the four largest and most comprehensive library databases: Pubmed, Embase, Web of Science and PsychInfo. Given the increasing attention that has been placed on gender disparities and health in recent years, we focused on studies published between 2010 and 2021.

**Results:**

Our search yielded 5,947 articles and 1,258 were IPV and SSA related. Out of this, only ten examined the intersection between IPV and TBI. All focused on outcomes in female populations from South Africa (*n* = 5), Ghana (*n* = 3), Uganda (*n* = 1), and Cameroon (*n* = 1). They were a mix of qualitative studies (*n* = 3), neuro-imaging/biomarker studies (*n* = 3), case studies/reports (*n* = 2), quantitative surveys (*n* = 1) and mixed qualitative/quantitative study (*n* = 1). Six studies evaluated subjective reporting of IPV-induced TBI symptoms such as headaches, sleep disruptions, and ophthalmic injuries. Three examined objective assessments and included Hypothalamic-Pituitary-Adrenal (HPA) dysregulation detected by salivary cortisol levels, magnetic resonance imaging (MRI) including diffusion tensor imaging (DTI) to evaluate brain connectivity and white matter changes. One final study took a forensic anthropology lens to document an autopsy case report of IPV-induced mortality due to physical head and face trauma.

**Conclusion:**

Our findings demonstrate that both subjective and objective assessments of IPV and TBI are possible in “resource-limited” settings. The combination of these outcomes will be critical for viewing IPV through a clinical rather than a cultural lens, and for substantiating the assertion that *gender*, is indeed, *a social determinant of brain health*.

## Background

Whereas, “biological sex” is defined as the relatively unchanging biology of being male or female, “gender” refers to the roles and expectations attributed to men and women in a given society (1). Being able to bear a child, for example, is fundamentally a function of biology, whereas expectations about the imperative to bear children, the nature of parenting, or the status associated with being a mother are more closely linked to gender roles and expectations (1). Recently, the literature on “gender as a social determinant of health” has focused on women because population-level data shows that on aggregate, women suffer more negative health consequences of inequalities between the sexes than men (1). Among these negative implications are intimate partner violence (IPV) and traumatic brain injury (TBI) (2–4).

TBI, defined as “an alteration in brain function or other evidence of brain pathology, caused by an external force” such as a blow or injury to the head, severe rotation of the neck, and acceleration/deceleration movement (5), is one of the leading causes of mortality and disability worldwide (6). TBI is estimated to affect 27 million people worldwide each year, with a disproportionately high incidence as well as prevalence in low- and middle-income countries (LMICs) (3, 4). Intimate Partner Violence (IPV), defined as behaviors by an intimate partner or ex-partner that causes physical, sexual, or psychological harm, including physical aggression, sexual coercion, psychological abuse, and controlling behaviors (2), is one of the leading, yet overlooked causes of TBI amongst women (7–9). Evidence shows that one in three women globally report physical IPV, and up to 92% experience blows to the head, face, or neck, making IPV victims incredibly susceptible to TBI morbidity (7). IPV-related TBI may have long-term impacts on a survivor's overall function and independence and can be misdiagnosed as a variety of other physical, social, and mental health conditions; making it difficult for survivors to get the help they need from health and community providers (9, 10). The prevalence of IPV in sub-Saharan Africa (SSA) is estimated to be one of the highest globally (11), with a lifetime prevalence of 33% [compared to the four subregions of Europe (16–23%), Central (18%), Eastern (20%) and South-Eastern Asia (21%), and Australia and New Zealand (23%)] and a 12-month prevalence of 20% [compared to Australia and New Zealand (3%), Northern America (6%), Eastern Asia and the subregions of Europe (4–7%)]. Yet, little is known about the association between IPV and TBI in the SSA context.

Current studies have identified sequelae of cognitive dysfunction, posttraumatic stress disorder, and depression in women experiencing IPV, yet, most fail to determine the role of TBI in the onset and propagation of these disorders (10). We use this call for papers on “Advancing the Representation of Minoritized Groups and Social Determinants of Health in Brain Injury Research” to examine the intersection between IPV and TBI in SSA, and yielding to the call to increase TBI research in SSA to better manage the care and treatment of patients in this part of the world (12). By conducting a scoping review of the existing body of research on the association between TBI and IPV in SSA, we aim to provide further evidence for establishing “gender as a social determinant of *brain* health” for a subgroup of the world's population who may be most vulnerable to TBI *via* gendered violence.

## Methods

We chose a scoping review for this endeavor to provide a preliminary overview of the existing gaps in the literature. Though both scoping reviews and systematic reviews use rigorous and transparent methods, scoping reviews seek to provide preliminary assessments of the extent, range, and nature of research activity on a topic; whereas systematic reviews are less exploratory, and are intended to sum up the best available research (13).

We used the following 5 steps recommended by Arksey and O'Malley (14), Pelaccia et al. (15), and De Allegri et al. (16):

In step 1, we identified the research question:

Which was to evaluate the literature on IPV in SSA and review those bodies of work for mentions of, or connections to TBI and/or brain imaging. Given the increasing attention that has been placed on gender disparities and health in recent years, we focused on studies published between 2010 and 2021.

In step 2, we identified relevant studies:

Our search was designed to capture research that explored the topic of IPV in SSA and TBI in peer-review journals. With the assistance of a librarian (LH), the first author (MAO) initiated a comprehensive search of bibliographic databases [with citation tracking using a combination of keywords and controlled vocabulary (**Appendix**)]. We used the four largest and most comprehensive library databases for this purpose: Pubmed, Embase, Web of Science, and PsychInfo.

Studies were extracted and managed in the EndNote bibliographical software (Copyright 2020 Clarivate Analytics) (17), using the following inclusion criteria:

1. Primary research papers published in 2010 or afterward
2. In the English language, that
3. Employ qualitative, quantitative, or mixed-method approaches to
4. Focus on IPV in SSA (to get a sense of the research effort invested in this subject matter) and
5. Evaluate TBI or TBI-related physical (e.g., eye injury, head bruises/contusions) or somatic (headache, sleep) sequelae as a consequence of IPV, or neuroimaging/biomarker assessments in the context of IPV.

We excluded (1) secondary papers (i.e., systematic reviews, scoping reviews, and narrative reviews), (2) studies that did not center on IPV such as those that were mainly on emotional/sexual violence and HIV/AIDS, (3) studies published in languages other than English, and (4) studies conducted outside SSA.

In step 3, we selected studies to be included in the review:

MAO and AG iteratively reviewed the title and abstract of each article that was abstracted to ensure that the papers met the inclusion criteria. In the presence of conflicts, adjudication was sought through discussion and involvement of co-authors as necessary. To ensure objectivity in the assessment process, the authors did not review eligible studies they authored. In consensus with the co-authors, the studies to be included in the final review were discussed and approved before the records were charted in Step 4 using the Preferred Reporting Items for Systematic Reviews and Meta-Analyses—Extension for Scoping Review (PRISMA-ScR) (18). Following that, the authors used a similar approach to review the full text of the selected articles for relevance.

In step 4, we charted the data:

We used a table in Microsoft Word to organize and categorize relevant information from the studies retained for the review. We identified categories such as year of publication, author(s), study aims, approach and research design, study population and country of focus, and findings. The co-authors reviewed the data charts to ensure accuracy as a form of inter-coder reliability to ensure consistency and validity.

In step 5, we collated, summarized, and reported the results:

We worked as a team to synthetize the findings iteratively. In particular, MAO, RGA, and AG took the responsibility to iteratively appraise the quality of the studies reviewed based on the information on the study population, study design, outcome assessment, and analysis. Guided by the PRISMA guidelines for reporting scoping reviews (18), the co-authors engaged in a series of interactive discussions about the relevance of the retained studies to the overarching research question and project goals.

## Results

**Figure 1:**
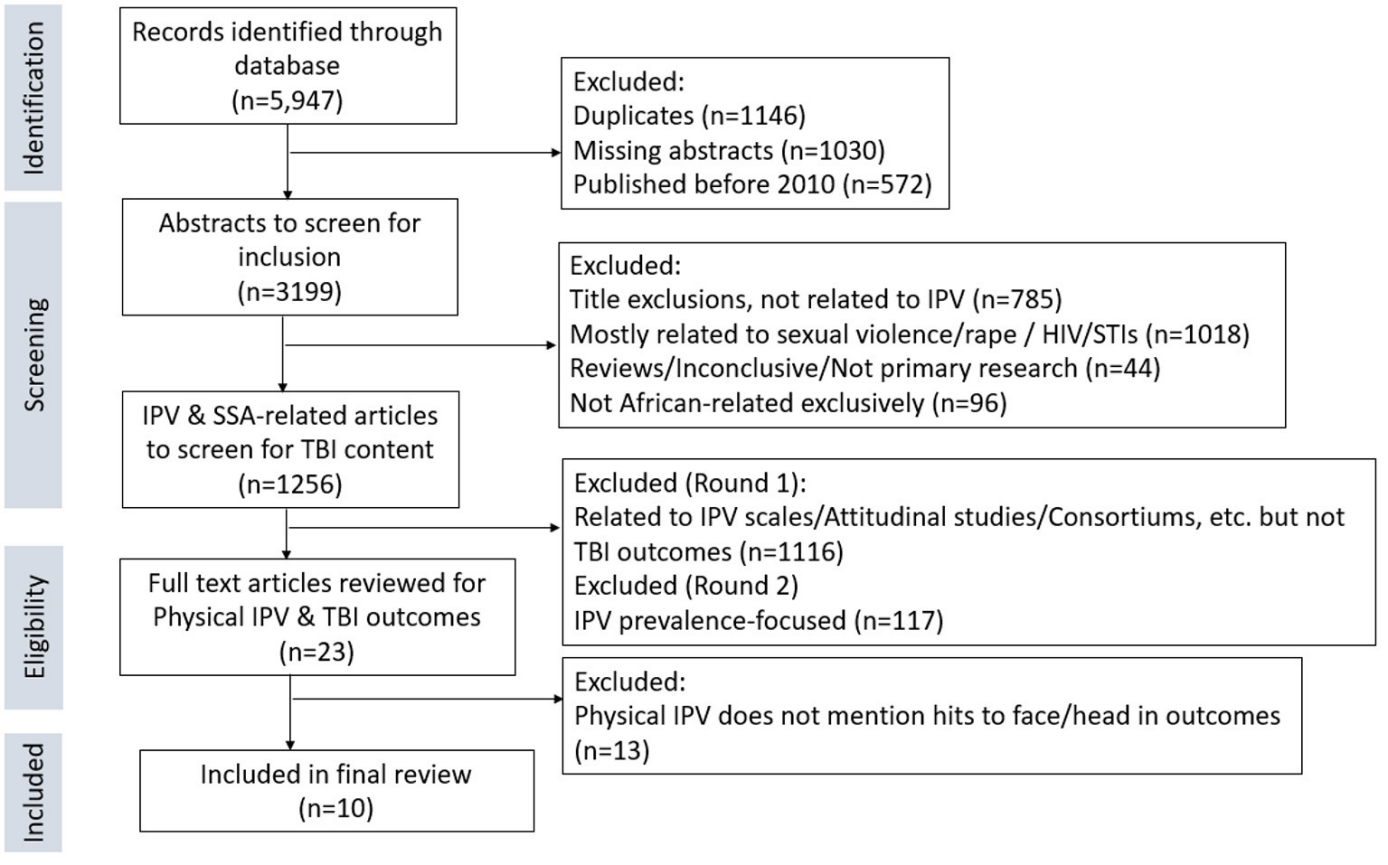
Flow chart of steps used in the selection of relevant studies.

The initial search identified 5,947 records from the databases (Figure 1). We excluded 1,146 duplicates, 1,030 studies with missing abstracts, and 572 articles published before 2010. We reviewed the abstracts of the remaining 3,199 records for relevant content. Another 2,748 were excluded, of which 785 were not related to IPV based on the title, 1,018 focused on sexual violence/rape/dating violence, 44 were reviews/not primary research/comments/inconclusive, and 96 were not exclusive to SSA.

The remaining 1,256 were all IPV and SSA related, but the majority were not associated with TBI outcomes. We had 2 rounds of evaluation for these articles. In the first round, we excluded any that were mainly focused on IPV scales, interventions, and attitudes/perceptions (*n* = 1,116). In Round 2, we excluded any that were just focused on prevalence (*n* = 117); leaving 23 articles for full-text review. For these final 23 articles, we assessed for mentions of physical trauma to the head or face and/or relevant TBI/brain imaging outcomes. We were left with 10 articles to include in the final review after excluding 13 that did not meet these criteria.

The 10 studies included in the final scoping review are summarized in Table 1. They were heterogeneous in design, and included qualitative studies (*n* = 3), neuro-imaging/biomarker studies (*n* = 3), case studies/reports (*n* = 2), quantitative surveys (*n* = 1) and mixed qualitative/quantitative methods (*n* = 1). All studies included female populations from South Africa (*n* = 5), Ghana (*n* = 3), Uganda (*n* = 1), and Cameroon (*n* = 1). The studies spanned the years 2010 to 2021, and included subjective reporting of physical-IPV induced TBI symptoms such as headaches (19, 21, 25), sleep disruptions (21–23, 25), and ophthalmic injuries (23, 25).

**Table 1:** Summary of studies that have focused on IPV in SSA and TBI-related outcomes (2010–2021); *n* = 10.

**Publication Year**	**Title and authors**	**Aims**	**Approach/study design**	**Population/country of focus**	**IPV and TBI-specific findings**
2010	Intimate partner violence, health behaviors, and chronic physical illness among South African women J. D. Gass, D. J. Stein, D. R. Williams and S. Seedat (19)	To study the association between physical intimate partner violence and physical health outcomes and behaviors among South African women.	Quantitative analyses of the cross-sectional, nationally representative South African Stress and Health Study	1,229 married and cohabiting South African women.	The prevalence of reported violence was 31%. Abused women reported higher odds of headache (OR 1.35; 95% CI: 0.98–1.88; *p* = 0.07) and neurological problems (OR 1.62; 95% CI: 0.51, 5.41; *p* = 0.41)
2011	“The neural correlates of intimate partner violence in women”: Erratum S. Flegar, J. Fouche, E. Jordaan, S. Marais, B. Spottiswoode, D. Stein, et al. (20)	To examine hippocampal volume and white matter tracts in women with and without intimate partner violence (IPV).	Women underwent structural magnetic resonance imaging (MRI) including diffusion tensor imaging (DTI) sequences.	19 women with IPV exposure in the last year, and 21 women without IPV exposure in the last year recruited from a rural community north of Cape Town, South Africa	IPV subjects did not demonstrate significantly different hippocampal volumes compared to subjects without recent IPV. Fractional anisotropy however, was significantly reduced in the body of the corpus callosum of IPV subjects
2012	Recognizing intimate partner violence in primary care: Western Cape, South Africa K. Joyner and R. Mash (21)	To evaluate how women experiencing IPV present in primary care, how often IPV is recognized by health care practitioners and what other diagnoses are made.	Three qualitative focus group interviews were held with healthcare practitioners and interviews with the facility managers to explore their experience of screening 114 medical records for IPV after being trained	Health care practitioners, namely all doctors and nurses at two urban and three rural community health center in South Africa	After screening 114 charts, IPV was previously recognized in 11 women (9.6%). Index of suspicion for IPV: headache, sleep disturbance
2015	Health implications of partner violence against women in Ghana P. A. Issahaku (22)	Explored health implications of partner violence against women in Northern Ghana	Mixed methods combined face-to-face structured interviews and regression analyses	443 women contacted at health facilities in the northern region.	7 out of 10 women have experienced IPV within the past 12 months. Physical IPV was associated with 4.82 times higher odds of sleep disruptions (95% CI: 2.38, 9.76; p=0.000)
2016	“… he always slaps me on my ears”: the health consequences of intimate partner violence among a group of patrilineal women in Ghana A. P. Sedziafa, E. Y. Tenkorang and A. Y. Owusu (23)	To explore the health effects of intimate partner violence among 15 ever-partnered Ghanaian patrilineal women.	Qualitative in-depth interviews	15 Ghanaian women of patrilineal lineage	Abused women reported sleeplessness and eye injuries, incurred from slaps and hits to the face
2017	Brain network connectivity in women exposed to intimate partner violence: a graph theory analysis study A. Roos, J. P. Fouche and D. J. Stein (24)	To explore how brain connectivity may be altered in individuals with IPV, but without PTSD.	Structural brain imaging using a Siemens 3T MRI. Global and regional brain network connectivity measures were determined, using graph theory analyses.	South African women exposed to IPV (*n* = 18) and healthy controls (*n* = 18)	Findings revealed altered connectivity on a global and regional level in the IPV group of regions involved in cognitive-emotional control, with principal involvement of the caudal anterior cingulate, the middle temporal gyrus, left amygdala and ventral diencephalon that includes the thalamus.
2021	Suffering in the Hands of a Loved One: The Endemic to Intimate Partner Violence and Consequences on Migrant Female Head-Load Carriers in Ghana E. B. Adomako and F. D. Baffour (25)	To evaluate the consequences of IPV on victims.	Qualitative interview with 20 women	Ghanaian women in the head-load carrying industry	Among the health-related effects of IPV reported by the subjects were eye injuries from slaps to the face, chronic headaches, and sleep deprivation
2021	Traumatic brain injury and forensic evaluations: Three case studies of U.S. asylum-seekers A. Saadi, P. Anand and S. L. Kimball (26)	To explore how to approach the forensic evaluation of asylum-seekers with a history of traumatic brain injury, illustrating the range of etiologies and sequelae of traumatic brain injury in this complex population.	Used three case vignettes	Foreign-born United States asylum seekers. We focused on a case study from Uganda for this review	A female case reported multiple episodes of head trauma, including two episodes of loss of consciousness lasting 2–15 min. On one occasion, she had direct trauma to her left orbit and had sustained symptoms of eyelid heaviness following this episode. These episodes left her with significant cognitive deficits, including memory difficulty, decreased concentration, and difficulty solving problems. On arrival to the US, she reported poor sleep (3 h on average per night, with nightmares when she did sleep). She was seen in ophthalmology, where she was diagnosed with a left -sided posterior vitreous detachment.
2021	HPA-axis activity and the moderating effect of self-esteem in the context of intimate partner violence in Cameroon D. L. Wadji, C. Gaillard, G. J. M. Ketcha Wanda, C. Wicky, N. Morina and C. Martin-Soelch (27)	Investigated how the IPV experienced by women in Cameroon affects their stress levels and those of their children	Three cortisol The total cortisol secretion over the first hour after awakening was determined by calculating the area under the curve with respect to the ground (AUCg).	IPV-exposed and control groups through the non-for-profit group Association for the Fight against Domestic Violence (ALVF) in Cameroon	Mothers exposed to IPV exhibited higher total post-awakening cortisol concentrations compared with those in the control group but not their children.
2021	Patterning of fractures in a case of intimate partner homicide (IPH) M. Steyn, N. Bacci and S. Holland (28)	Evaluate case of intimate partner homicide in a forensic anthropological context	Case Study	South African woman	Physical IPV-induced mortality due to trauma to the head and face after reportedly being hit by a brick, evidenced by perimortem fractures of the face

By design, the qualitative studies were the most descriptive and explicit, citing participants' accounts of their experiences with IPV as elaborated by Adomako et al. (25).

“*My partner slapped me one night and for one week my eyes turned to red” (participant Abena) “I had to remain indoors for some days because I had blood clots on my eyes after my husband had beaten me. I suffered injuries on my face with my eyes being the most affected.” (participant Fati)*

The vignettes by Saadi et al. (26) also provided great detail on the participants' IPV and TBI experiences, referencing concussion-like symptoms, ophthalmic injuries, consequential cognitive dysfunction, and the patients' coping mechanism. As detailed:

“*[the female Ugandan asylum seeker] reported multiple episodes of head trauma, including two episodes of loss of consciousness that she believes lasted between 2 and 15 min. On one occasion, she had direct trauma to her left orbit and had sustained symptoms of eyelid heaviness following this episode. These episodes left her with memory difficulty, decreased concentration, and difficulty solving problems. On arrival to the United States, she reported poor sleep…3 hrs on average per night, with nightmares when she did sleep…she report[ed] that if she were denied asylum, she would rather harm herself than be deported…she reported her religious faith and prayer as resiliency factors…she was seen in ophthalmology, where she was diagnosed with a left-sided posterior vitreous detachment.”*

Though the neuroimaging and biomarker studies were not as descriptive, they were comprehensive and included evaluations of Hypothalamic-Pituitary-Adrenal (HPA) dysregulation detected by salivary cortisol levels (27), magnetic resonance imaging (MRI) including diffusion tensor imaging (DTI) to evaluate brain connectivity and white matter changes (20, 24). The authors assessed several anatomic features of the brain including the corpus callosum, the caudal anterior cingulate, the middle temporal gyrus, the left amygdala, and ventral diencephalon, including the thalamus.

One final study was an autopsy report of IPV-induced mortality due to trauma to the head and face (28). In this case report, the authors used a forensic anthropology lens to issue a call to the medical fraternity and victims' social networks about the urgency of chronic abuse. As they state,

“*The deceased had suffered massive, repeated trauma during her lifetime with healed fractures and evidence of soft tissue trauma to virtually all parts of her body. A partly healed rib fracture indicates that the abuse continued until shortly before her death. She ultimately succumbed after suffering trauma to her head and face after reportedly being hit by a brick… the evidence suggests a very long period of extreme and repeated trauma, which were apparently not reported or noticed by family members or the medical fraternity.”*

Across almost all the studies, the authors make a connection between IPV-induced TBI and health-related support services for victims. The teams of Gass (19), Joyner (21), Issahaku (22), Sedziafa (23), Saadi (26), and Wadji (27) all discuss the need to either increase identification through health-related encounters or improve health-related services to better support victims. In investigating the association between IPV and health seeking behaviors, Gass et al. (19) found that compared with non-abused women, women reporting IPV were 1.5 times more likely to have visited a physician/healthcare facility and nearly twice as likely to have visited a traditional healer in the past 12 months. Wadji et al. (27) capitalized on the care-seeking nature of IPV victims to investigate the effect of IPV on HPA-axis activity in mother–child dyads exposed to IPV in Cameroon. Knowing that victims seek help, they partnered with the Association for the Fight against Domestic Violence (ALVF), a non-profit organization that provides free legal advice, guidance, and support to women victims of IPV to establish an association between IPV and serum cortisol concentrations. Participants were eager and compliant, resulting in a well-powered and rigorously designed study that not only showed that biomarker studies are feasible in non-Western settings for extremely vulnerable populations, but also established a biological and causal association between IPV and HPA dysregulation. The approach of Saadi et al. (26) was much more systematic. The authors (1) first make reference to the high prevalence of TBI amongst refugees and asylum seekers to the United States, (2) proceed to acknowledge IPV as a mechanism of TBI, (3) acknowledge the short and long term sequelae of IPV-induced TBI by noting the three symptom domains (somatic: headaches, sleep disruptions, dizziness, nausea, visual disturbance, photophobia, and phonophobia; cognitive: increased distractibility, slow processing speed, and difficulty concentrating or multitasking; and affective: increased irritability, emotional lability, anxiety, or depression), then (4) proceed to highlight the critical role of clinicians in connecting patients' IPV history with their TBI symptomatology and clinical presentations. The authors recommend that clinicians use TBI specific tools (e.g., Rivermead Post-Concussion Symptom Questionnaire, Neurobehavioral Symptom Inventory, etc.) for classification (mild/moderate/severe TBI) and assessment of TBI chronicity; and note that clinicians should be aware of the role of gender in brain health; an argument supported by Gass el al.'s (19) statement that IPV is a leading cause of morbidity and mortality for South African women, and over half of female homicide victims in South Africa are killed by their intimate partners. That IPV victims interact more with healthcare systems than non-abused women (19) emphasizes the need to “normalize” IPV screening; particularly in the SSA context. In all 10 papers included in the review, the authors acknowledged the over-abundance of IPV-related studies across the literature; particularly in the SSA context, but a lack of attention to specific, anatomic and/or neurological consequences of IPV. By these accounts, these 10 papers (out of a possible 1,258) were/are ahead of the curve.

## Discussion

Given that SSA has one of the highest IPV morbidity globally (11), this region of the world offers ample opportunity for TBI researchers to explore deeper associations between TBI and IPV to better understand the role of *gender* in TBI research. Our scoping review aimed to identify IPV-related studies that have explored the intersection of TBI-related outcomes in the SSA context. Out of a possible 1,258 studies that were IPV and SSA related, only 10 met our inclusion criteria and focused on TBI and/or brain imaging/biomarkers in the context of IPV. The majority of studies described IPV prevalence, attitudinal views of IPV amongst healthcare providers and other relevant populations, or focused on IPV screeners and interventions, but rarely detailed the physical impact of IPV on the victims; particularly TBI. Even though TBI can result from IPV, the connection between the two is often missed-THUS the impetus of our paper. This disconnect between IPV and TBI is common, even in “Western” settings (29, 30); including amongst service providers (31). Had we chosen to only include papers that explicitly made the connection between IPV and TBI, we would not have this manuscript. Of the 10 papers included in the review, other than Saadi et al. (26) and Steyn et al. (28) explicitly mentioning “head trauma,” the remainder referred to symptoms or sequelae commonly experienced by IPV victims and reported across the wider literature (7, 10) including headaches (19, 21, 25), sleep disruptions (21–23, 25), and ophthalmic injuries (23, 25).

The reasons for the disconnect are multitude. A study by Sheridan and Nash (32) outlined the difficulty in doing research on the “acute injury patterns of IPV victims” due to the mix of medical and non-medical terminology used in provider reports and patient medical records (e.g., “busted lip” instead of “laceration”). In addition, there is heterogeneity in search terms, as medical documentation describes the elements of acute trauma as symptoms or observations, such as “lacerations,” “facial fractures,” or “mental status change” and not the sequelae of these, which is the “traumatic brain injury.” Determining prevalence across studies is also difficult due to differences in screening protocols (33), location difference (health clinic vs. Integrated health delivery system), survey methods (anonymous vs. Clinic screening), definition of IPV (emotional, physical, sexual, psychological), mechanism of injury (blunt force, strangulation) and timeframe (lifetime vs. recent) (32, 34, 35).

These limitations pose great challenges for researchers attempting to connect the IPV and TBI dots. As noted by Gass et al. (19) and Steyn (28), IPV is associated with increased use of medical services. However, there is reticence on the part of healthcare professionals to investigate IPV-related injuries due to provider fear of offending the patient or their partner (particularly if a negative screen), provider fear of exacerbating the problem/retaliation for the patient (if a positive screen), providers' perceived lack of power to change the problem, cultural differences, and overall provider discomfort with IPV (35–39). These perceived barriers are universal.

In a survey of Nigerian healthcare providers for example, 6 in 10 deemed it “an intrusion into a patient's private life” to inquire about IPV exposure, and 9 in 10 expressed concern for their personal safety if they were to discuss with the oppressed or perpetrators (40). Providers opted instead to “turn a blind eye” and avoid the “elephant in the room” if a patient was suspected victim. In fact many African scholars writing on domestic violence emphasize battering and domestic violence as falling under the rubric of “culture” rather than offering objective, clinical explanations (41). Patients who are screened for IPV however, report greater satisfaction with their care (42). *We must move beyond accepting IPV as a problem of culture, shrouding it in reticence; and move toward the clinical and pathological ramifications of gendered violence*.

Similarly to the results found in our review about health-related support services for victims of IPV-induced TBI, other authors have noted the need for a health-based approach to identification and care of this population. Recently, researchers have focused on IPV screening in the Emergency Department (ED), which often serves as the first source of care for many victims. The burgeoning growth of emergency medicine (EM) training programs across the African region (43–46) presents timely and ample opportunities to “shift” the lens through which IPV is viewed across the continent and to “normalize” IPV screening, diagnosis and treatment. Providers should be trained to evaluate suspected IPV patients using TBI protocols that include (but are not limited to) utilizing neuroimaging procedures to identify intracranial injuries that require (1) immediate surgical or procedural intervention, (2) medical therapy or vigilant neurologic supervision, or (3) prognostication of patients to tailor rehabilitative therapy, counseling or discharge home (47). Non-contrast CT (NCCT) is the initial triaging diagnostic imaging test of choice for patients with TBI (48). NCCT is sensitive and specific for the presence of intracranial hemorrhage, extra-axial fluid collections, skull fractures, cerebral edema, swelling and signs of herniation. NCCT has many advantages, including its widespread availability and rapid acquisition time, with few contraindications (47). And unlike magnetic resonance imagining (MRI), NCCT does not require screening of patients for ferromagnetic substances such as metal or cardiac pacemakers and other contraindicated implantable devices and materials before scanning (47). Neuroimaging tools can be combined with HPA biomarkers (27) and the aforementioned TBI specific tools (Rivermead Post-Concussion Symptom Questionnaire, etc.) to identify and prognosticate IPV victims. As has been previously reported in the literature, elevated cortisol levels are observed in patients who experience IPV (49), which might be explained by the impact of IPV-related-TBI on the HPA axis physiologic function (50). Also, existing studies in the broader literature have examined the possibility of using cortisol levels as biomarkers for neuropsychiatric IPV sequelae, such as PTSD (49). Thus, this set of biomedical findings draws attention to the issue that the interchangeable relation between IPV and TBI might be a leading cause of detrimental long-term complications that are often overlooked (51, 52). The effectiveness and reliability of IPV-induced TBI clinical screening protocols might be improved by implementing similar imaging and testing modalities (53). To systematically and continuously connect the IPV and TBI dots, utilization and training of brain imaging techniques and biomarker diagnostic tools should be supplemented with IPV and TBI-specific modules that are integrated across various “educational intervention points” at the undergraduate, internship/housemanship, practitioner/attending, and professional development levels for EM providers (45).

Typically, the reaction from researchers and implementation scientists is to deem diagnostic tools like neuroimaging as too “resource-heavy”, and challenging- from economic, training, and/or sustainability perspectives-for low-resource settings. But our review findings demonstrate otherwise. The heterogeneous nature of the ten studies we identified, particularly those that included objective biomarkers and neuroimaging tools, point to a “future of possibilities”, suggesting that neuroimaging assessments of IPV in the clinical setting are indeed, possible in “resource-limited” settings. In fact, Flegar et al. and Roos et al. (20, 24) show that MRI and DTI techniques *can be* utilized with IPV patients in clinical settings in SSA to demonstrate pathology; and the feasibility of neuroimaging in brain disorders has been demonstrated in several clinical contexts across the region (48, 54, 55); with providers inventing innovative alternative approaches when faced with resource limitations. For example, limited access to helium and the infrastructure necessary to support superconducting magnets in Nigeria spurred the development of commercially available, low-field permanent magnet MR systems (48); offering providers the decision-making tools they need to make diagnoses, and patients the appropriate care needed.

**Figure 2:**
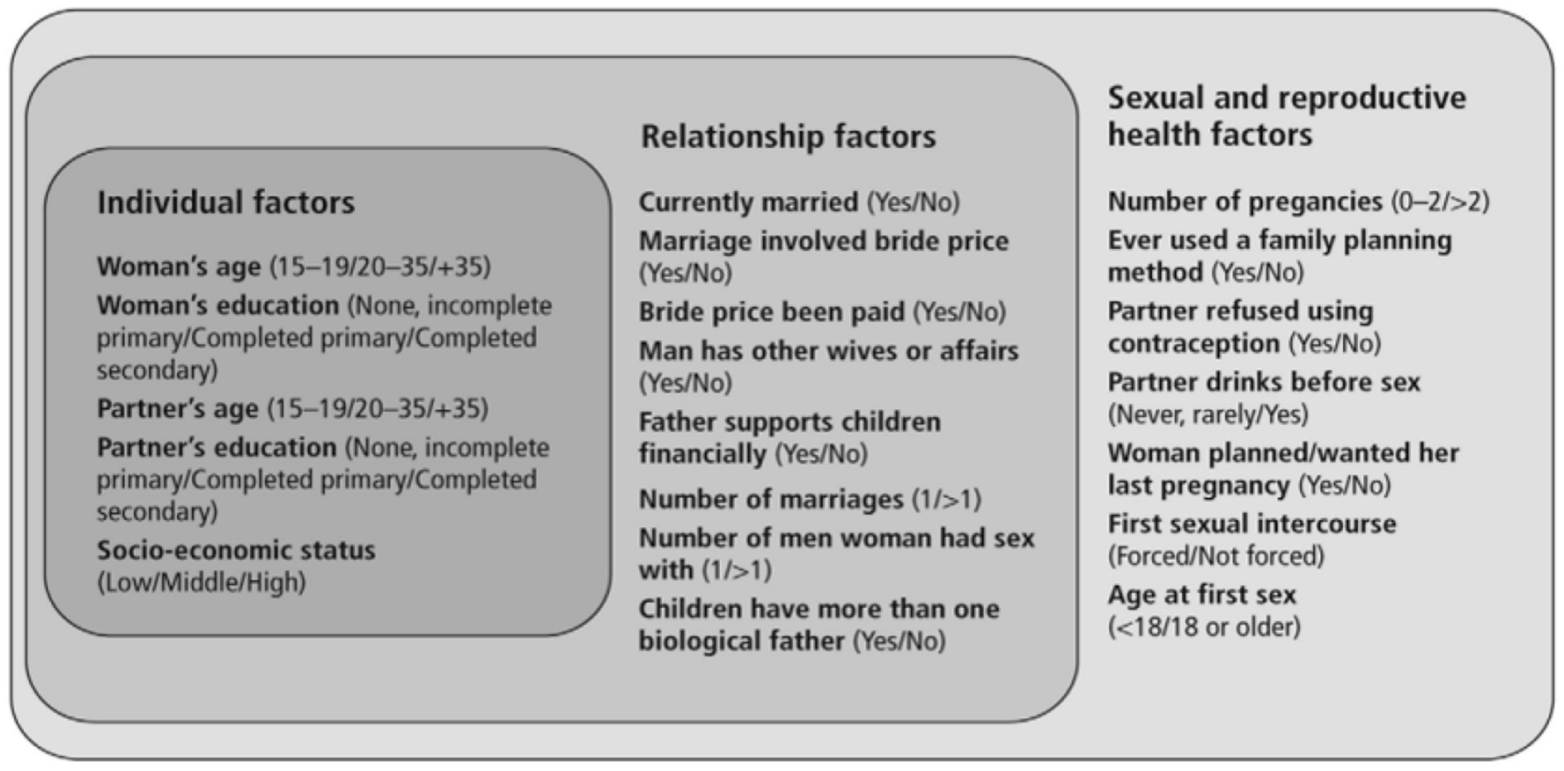
Conceptual framework of potential individual, relationship and sexual and reproductive health factors that may be associated with violence during pregnancy, outlining the measurement of factors analyzed in this study. Adapted from Stöckl et al. (58).

It is imperative that researchers integrate IPV and TBI-related outcomes with objective assessments before the *gendered norms of culture* cause more women to become cases for forensic anthropologists. Additionally, research that combines subjective IPV reporting (by patient or provider) with objective, clinical neuroimaging tools will be critical for viewing IPV through a clinical, rather than a cultural lens; and for substantiating *gender as a social determinant of* brain *health*. Such research initiatives could be integrated with collaboratives such as the Enhancing NeuroImaging Genetics through Meta-Analysis (ENIGMA) Consortium Intimate Partner Violence Working Group (56), which aims to develop a global network of researchers, clinicians, and stakeholders, working on the neurobehavioral and neurobiological effects of head trauma in the IPV population. Biomarkers and neuroimaging methods could be integrated with ongoing efforts to harmonize data collection and analyses efforts across the various sites within the working groups, to characterize how intimate partner violence-related head trauma impacts long-term physical, cognitive, psychological, and even reproductive/sexual health. Time to event analyses of 14 SSA countries revealed that the median time to first spousal violence after marriage in Western, Middle, Eastern, and Southern Africa was 2 years (57). To further evaluate the association between IPV, TBI, and women's reproductive/sexual health, conceptual frameworks such as those proposed by Stöckl et al. (58) (Figure 2) could provide guidance on the most relevant “assessment timepoints” for research and intervention (e.g., targeting socioeconomic status at the individual factor level vs. relationship factors).

We hope that through this work, researchers will be “called to action” to recognize and address the elephant in the room; that when a patient is a suspected victim of IPV, TBI should not be too far off in thought; that by making this connection, IPV can be viewed through a clinical lens.

## Strengths and limitations

To the best of our knowledge, this is the first study to evaluate the intersection of IPV and TBI-related in the SSA context. Our search, however, focused on English-only papers and may have inadvertently missed publications that reflect IPV morbidity in Francophone and Portuguese Africa. However, we cast a broad net by searching four of the largest and most comprehensive library databases (Pubmed, Embase and Web of Science and PsychInfo), resulting in almost 6,000 “hits”. Future research should broaden the search criteria to include manuscripts from that part of the global south.

To the authors' knowledge, there is limited (comparative) literature on the impact of IPV on Cis vs. Trans-gendered individuals; particularly in the SSA context. A systematic review of the literature showed that transgender individuals experience a dramatically higher prevalence of IPV victimization compared with cisgender individuals (59); though this was limited to a mostly United States (US) population. The inclusion/exclusion criteria for this work was not limited to the cis-gender or female literature exclusively and thus would have yielded publications across SSA that compared and/or included transgendered persons, as well as men who experience IPV. IPV however, is highly gendered and stigmatized and male/non-cis IPV is underrepresented across the literature. Research that focuses on these overlooked populations; particularly in the SSA context is needed to fully understand the landscape of IPV in highly normed settings.

## Conclusions

Despite the over-abundance of IPV-related studies across the literature in the SSA context, there is a paucity of attention to specific, anatomic and/or neurological consequences of IPV. The inclusion of objective assessments of the neurological impact of IPV in SSA in this scoping review demonstrates that objective biomarker assessments of IPV are possible in “resource-limited” settings. The combination of these biomarkers and subjective reporting will be critical for viewing IPV through a clinical lens rather than a cultural lens, and for sustaining the assertion that *gender, is indeed, a social determinant of brain health*.

## Author contributions

MA-O and RA: conceptualization. LH, MA-O, and AG: data curation. MA-O and AG: formal analysis. MA-O, AG, and RA: investigation and validation. MA-O, RA, and LH: methodology. MA-O: project administration, resources, supervision, visualization, and writing-original draft. MA-O, RA, LH, AG, SO-A, and RB: writing-review and editing. All authors contributed to the article and approved the submitted version.

## Funding

At the time of press, MA-O was funded by NIH NINDS K01 Award#7K01NS121199-02.

## Conflict of interest

The authors declare that the research was conducted in the absence of any commercial or financial relationships that could be construed as a potential conflict of interest.

## Publisher's note

All claims expressed in this article are solely those of the authors and do not necessarily represent those of their affiliated organizations, or those of the publisher, the editors and the reviewers. Any product that may be evaluated in this article, or claim that may be made by its manufacturer, is not guaranteed or endorsed by the publisher.
