# Supplementary material for: The elephant in the room: Intimate partner violence, women, and traumatic brain injury in sub-Saharan Africa
Source: Front Neurol. 2022 Sep 6;13:917967. doi: 10.3389/fneur.2022.917967 (PMC9485886; doi:10.3389/fneur.2022.917967)
Supplement: Supplementary file 1 [file Data_Sheet_1.docx]

# APPENDIX OF SEARCH TERMS

**PubMed**

(Intimate partner violence OR intimate partner abuse OR dating violence OR Spouse Abuse OR Spousal Abuse) AND (Angola OR Benin OR Botswana OR Burkina Faso OR Burundi OR Cameroon OR Cape Verde OR Central African Republic OR Chad OR Comoros OR Congo democratic republic OR congo Brazzaville OR Côte d'Ivoire OR Ivory Coast OR Djibouti OR Equatorial Guinea OR Eritrea OR Ethiopia OR Gabon OR Gambia OR Ghana OR Guinea OR Guinea-Bissau OR Kenya OR Lesotho OR Liberia OR Madagascar OR Malawi OR Mali OR Mauritania OR Mauritius OR Mozambique OR Namibia OR Niger OR Nigeria OR Reunion OR Rwanda OR Sao Tome and Principe OR Senegal OR Seychelles OR Sierra Leone OR South Africa OR Sudan OR Swaziland OR Tanzania OR Togo OR Uganda OR Western Sahara OR Zambia OR Zimbabwe OR Sub Saharan Africa OR Sub-Saharan Africa)

**Embase**

('africa south of the sahara'/exp OR 'africa south of the sahara' OR 'black africa' OR 'sub saharan africa' OR 'subsaharan africa') AND ('partner violence'/exp OR 'intimate partner violence' OR 'partner abuse' OR 'partner violence' OR 'spouse abuse' OR 'dating violence'/exp OR 'dating violence')

**Web of Science**

TS=(Intimate partner violence OR intimate partner abuse OR dating violence OR Spouse Abuse OR Spousal Abuse) AND TS=(Angola OR Benin OR Botswana OR Burkina Faso OR Burundi OR Cameroon OR Cape Verde OR Central African Republic OR Chad OR Comoros OR Congo democratic republic OR congo Brazzaville OR Côte d'Ivoire OR Ivory Coast OR Djibouti OR Equatorial Guinea OR Eritrea OR Ethiopia OR Gabon OR Gambia OR Ghana OR Guinea OR Guinea-Bissau OR Kenya OR Lesotho OR Liberia OR Madagascar OR Malawi OR Mali OR Mauritania OR Mauritius OR Mozambique OR Namibia OR Niger OR Nigeria OR Reunion OR Rwanda OR Sao Tome and Principe OR Senegal OR Seychelles OR Sierra Leone OR South Africa OR Sudan OR Swaziland OR Tanzania OR Togo OR Uganda OR Western Sahara OR Zambia OR Zimbabwe OR Sub Saharan Africa OR Sub-Saharan Africa)

**PsycINFO**

| 1. | intimate partner violence.mp. or exp Intimate Partner Violence/ |
| --- | --- |
| 2. | spousal abuse.mp. |
| 3. | exp Dating Violence/ |
| 4. | dating violence.mp. or exp Dating Violence/ |
| 5. | 1 or 2 or 3 or 4 |
| 6. | sub-saharan africa.mp. |
| 7. | Angola.mp. |
| 8. | Benin.mp. |
| 9. | Botswana.mp. |
| 10. | Burkina Faso.mp. |
| 11. | Burundi.mp. |
| 12. | Cameroon.mp. |
| 13. | Cape Verde.mp. |
| 14. | Central African Republic.mp. |
| 15. | Chad.mp. |
| 16. | Comoros.mp. |
| 17. | democratic republic congo.mp. |
| 18. | congo Brazzaville.mp. |
| 19. | ivory coast.mp. |
| 20. | Djibouti.mp. |
| 21. | Equatorial Guinea.mp. |
| 22. | Eritrea.mp. |
| 23. | Ethiopia.mp. |
| 24. | Gabon.mp. |
| 25. | Gambia.mp. |
| 26. | Ghana.mp. |
| 27. | Guinea.mp. |
| 28. | Guinea-Bissau.mp. |
| 29. | Kenya.mp. |
| 30. | Lesotho.mp. |
| 31. | Liberia.mp. |
| 32. | Madagascar.mp. |
| 33. | Malawi.mp. |
| 34. | Mali.mp. |
| 35. | Mauritania.mp. |
| 36. | Mauritius.mp. |
| 37. | Mozambique.mp. |
| 38. | Namibia.mp. |
| 39. | Niger.mp. |
| 40. | Nigeria.mp. |
| 41. | Reunion.mp. |
| 42. | Rwanda.mp. |
| 43. | Sao Tome Principe.mp. |
| 44. | Senegal.mp. |
| 45. | Seychelles.mp. |
| 46. | Sierra Leone.mp. |
| 47. | South Africa.mp. |
| 48. | Sudan.mp. |
| 49. | Swaziland.mp. |
| 50. | Tanzania.mp. |
| 51. | Togo.mp. |
| 52. | Uganda.mp. |
| 53. | Western Sahara.mp. |
| 54. | Zambia.mp. |
| 55. | Zimbabwe.mp. |
| 56. | Sub-Sahar* Africa.mp. [mp=title, abstract, heading word, table of contents, key concepts, original title, tests & measures, mesh word] |
| 57. | 7 or 8 or 9 or 10 or 11 or 12 or 13 or 14 or 15 or 16 or 17 or 18 or 19 or 20 or 21 or 22 or 23 or 24 or 25 or 26 or 27 or 28 or 29 or 30 or 31 or 32 or 33 or 34 or 35 or 36 or 37 or 38 or 39 or 40 or 41 or 42 or 43 or 44 or 45 or 46 or 47 or 48 or 49 or 50 or 51 or 52 or 53 or 54 or 55 or 56 |
| 58. | 5 and 57 |
